# *Candidatus* Neoehrlichia mikurensis and *Anaplasma phagocytophilum* in Urban Hedgehogs

**DOI:** 10.3201/eid2003.130935

**Published:** 2014-03

**Authors:** Gábor Földvári, Setareh Jahfari, Krisztina Rigó, Mónika Jablonszky, Sándor Szekeres, Gábor Majoros, Mária Tóth, Viktor Molnár, Elena C. Coipan, Hein Sprong

**Affiliations:** Szent István University, Faculty of Veterinary Science, Budapest, Hungary (G. Földvári, K. Rigó, M. Jablonszky, S. Szekeres, G. Majoros, V. Molnár);; National Institute of Public Health and Environment, Bilthoven, the Netherlands (S. Jahfari, E.C. Coipan, H. Sprong);; Hungarian Natural History Museum, Budapest (M. Tóth);; Budapest Zoo and Botanical Garden, Budapest (V. Molnár)

**Keywords:** *Candidatus* Neoehrlichia mikurensis, Anaplasma phagocytophilum, bacteria, northern white-breasted hedgehog, hedgehog, Erinaceus roumanicus, Ixodes ricinus, ticks, city park, urban hedgehog, Budapest, Hungary, Europe

**To the Editor:** *Candidatus* Neoehrlichia mikurensis is a member of the order Rickettsiales, family *Anaplasmataceae* (*1*). Manifestations of infection with these bacteria are atypical and severe and include cough, nausea, vomiting, anemia, headache, pulmonary infiltration, malaise, myalgia, arthralgia, fatigue, recurrent fever for ≤8 months, and/or death (*2**–**5*). *Candidatus* N. mikurensis has been detected in *Ixodes ovatus*, *I. persulcatus*, and *Haemaphysalis concinna* ticks in Asia (*1**,**5*).

*Candidatus* N. mikurensis has been identified as one of the most prevalent pathogenic agents in *I. ricinus* ticks throughout Europe (*2**,**3**,**6*). Rodents of diverse species and geographic origins have been shown to carry these bacteria, but transmission experiments have not been conducted to unambiguously identify natural vertebrate reservoirs (*1**–**3**,**5**–**7*). This emerging tickborne pathogen has been detected mainly in immunocompromised patients in Sweden (n = 1), Switzerland (n = 3), Germany (n = 2), and the Czech Republic (n = 2) and in immunocompetent patients in China (n = 7) (*2**–**5*).

*Anaplasma phagocytophilum* is an obligate, intracellular, tickborne bacterium of the family *Anaplasmataceae* and causes granulocytic anaplasmosis in humans and domestic animals. In Europe, *I. ricinus* ticks are its major vector, and red deer, roe deer, rodents, and European hedgehogs (*Erinaceus europaeus*) are suspected reservoir hosts (*8*).

Northern white-breasted hedgehogs (*Erinaceus roumanicus*) are urban-dwelling mammals (order Eulipotyphla, family Erinaceidae) that serve as major maintenance hosts for the 3 stages of *I. ricinus* ticks (*9*). However, *E. roumanicus* hedgehogs have not been studied for their ability to carry *A. phagocytophilum*. In addition, no suspected reservoirs other than rodents have been investigated for *Candidatus* N. mikurensis. The purpose of this study was to determine whether this hedgehog is a potential reservoir of these 2 bacteria.

We conducted an ecoepidemiologic study during 2009–2011 to obtain information about ticks and tickborne pathogens of urban hedgehogs in a park on Margaret Island in central Budapest, Hungary (*9*). Ear tissue samples were obtained from hedgehogs anesthetized with intramuscular ketamine (5 mg/kg) and dexmedetomidine (50 µg/kg).

DNA was extracted from samples by using the QIAamp DNA Mini Kit (QIAGEN, Hilden, Germany) or the Miniprep Express Matrix protocol (MP Biomedicals, Santa Ana, CA, USA). We used quantitative real-time PCRs that partially amplify the heat shock protein gene (*groEL*) of *Candidatus* N. mikurensis and the merozoite surface protein 2 gene (*msp2*) of *A. phagocytophilum* (*3*). PCR was performed in a 20-μL volume containing iQ Multiplex Powermix (Bio-Rad Laboratories, Hercules, CA, USA) in a LightCycler 480 Real-Time PCR System (F. Hoffmann-La Roche, Basel, Switzerland). Final PCR concentrations were 1× iQ Powermix, 250 nmol/L of primers ApMSP2F and ApMSP2R, 125 nmol/L of probe ApMSP2P-FAM, 250 nmol/L of primers NMikGroEL-F2a and NMikGroEL-R2b, 250 nmol/L of probe NMikGroEL-P2a-RED, and 3 μL of template DNA.

To confirm quantitative PCR results, we performed conventional PCRs in a Px2 Thermal Cycler (Thermo Electron Corporation, Waltham, MA, USA) on selected PCR-positive samples for both pathogens (*3*). Sequences obtained were submitted to GenBank under accession nos. KF803997 (*groEL* gene of *Candidatus* N. mikurensis) and KF803998 (*groEL* gene of *A. phagocytophilum*).

*Candidatus* N. mikurensis was detected in 2 (2.3%) of 88 hedgehog tissue samples. Formerly, rodents were the only wild mammals found to act as potential reservoirs for this pathogen. Results of studies that attempted to detect these bacteria in common shrews (*Sorex araneus*), greater white-toothed shrews (*Crocidura russula*) (*2**,**3*), or common moles (*Talpa europaea*) (*2*) were negative. However, our results indicate that northern white-breasted hedgehogs might be a non-rodent reservoir for *Candidatus* N. mikurensis.

The low pathogen prevalence observed in this urban hedgehog population compared with that in rodents in other locations (*2**,**3*) might be caused by use of skin samples. Skin samples from rodents showed only 1.1% positivity in a study in Germany; however, average prevalence of *Candidatus* N. mikurensis in transudate, spleen, kidney, and liver samples from the same animals was 37.8%–51.1% (*2*). Although we did not test other organs, we hypothesize that prevalence of *Candidatus* N. mikurensis infection urban hedgehogs is probably >2.3%.

We detected *A. phagocytophilum* in 67 (76.1%) of 88 urban hedgehogs. This prevalence was similar to that found among European hedgehogs in Germany (*8*). *I. ricinus* ticks are more common than *I. hexagonus* ticks in this urban hedgehog population (*9*). Thus, *I. ricinus* ticks can acquire these bacteria when feeding on hedgehogs and the risk for human infection with *A. phagocytophilum* in this park in Budapest is relatively high.

Neoehrlichiosis and granulocytic anaplasmosis have not been diagnosed in humans in Hungary. This finding is probably caused by diagnostic difficulties rather than absence of these pathogens in the environment. Infection with *Candidatus* N. mikurensis and *A. phagocytophilum* cause predominantly noncharacteristic symptoms. Laboratory cultivation and serologic detection of *Candidatus* N. mikurensis has not been successful, and this pathogen has not been identified in blood smears. Thus, accurate diagnosis of suspected cases requires suitable molecular methods.

Parks can be considered points of contact for reservoir animals, pathogens, ticks, and humans. Our results indicate that *E. roumanicus* hedgehogs play a role in urban ecoepidemiology of ≥2 emerging human pathogens. To better understand the urban cycle of these pathogens, potential reservoir hosts, ticks collected from these hosts, and vegetation in parks should be investigated.
